# Neural crest multipotency and specification: power and limits of single cell transcriptomic approaches

**DOI:** 10.12703/r/10-38

**Published:** 2021-04-14

**Authors:** Kristin B Artinger, Anne H Monsoro-Burq

**Affiliations:** 1 Department of Craniofacial Biology, University of Colorado School of Dental Medicine, Aurora, CO, USA; 2 Université Paris-Saclay, Faculté des Sciences d'Orsay, France; 3 Institut Curie, INSERM U1021, CNRS UMR3347, Orsay, France; 4 Institut Universitaire de France, Paris, France

**Keywords:** neural crest, multipotency, pluripotency, single cell transcriptomics, fate specification, lineage specification

## Abstract

The neural crest is a unique population of multipotent cells forming in vertebrate embryos. Their vast cell fate potential enables the generation of a diverse array of differentiated cell types *in vivo*. These include, among others, connective tissue, cartilage and bone of the face and skull, neurons and glia of the peripheral nervous system (including enteric nervous system), and melanocytes. Following migration, these derivatives extensively populate multiple germ layers. Within the competent neural border ectoderm, an area located at the junction between the neural and non-neural ectoderm during embryonic development, neural crest cells form in response to a series of inductive secreted cues including BMP, Wnt, and FGF signals. As cells become progressively specified, they express transcriptional modules conducive with their stage of fate determination or cell state. Those sequential states include the neural border state, the premigratory neural crest state, the epithelium-to-mesenchyme transitional state, and the migratory state to end with post-migratory and differentiation states. However, despite the extensive knowledge accumulated over 150 years of neural crest biology, many key questions remain open, in particular the timing of neural crest lineage determination, the control of potency during early developmental stages, and the lineage relationships between different subpopulations of neural crest cells. In this review, we discuss the recent advances in understanding early neural crest formation using cutting-edge high-throughput single cell sequencing approaches. We will discuss how this new transcriptomic data, from 2017 to 2021, has advanced our knowledge of the steps in neural crest cell lineage commitment and specification, the mechanisms driving multipotency, and diversification. We will then discuss the questions that remain to be resolved and how these approaches may continue to unveil the biology of these fascinating cells.

## Introduction

The advent of single cell RNA sequencing (scRNA-seq) (see Box 1) and single cell epigenomics approaches has raised immense expectations among developmental biologists. The hope is to "read" the developmental genetic program of each cell and deduce the rules of cell lineage progression by using cell-to-cell transcriptome heterogeneities to trace both spatial and temporal "pseudotime trajectories" (see Box 1 and Box 2) as well as mapping transcription factor binding sites to deduce developmental regulatory modules^1^.

**Box 1. Standard pipeline for single cell transcriptome analysis**

1- Cell dissociation

Whole embryos (Zebrafish, *Xenopus*) are dissociated into individual cells, and each cell is processed using droplet-based microfluidics methods (inDrops, 10X). Alternatively, neural crest cells are selected using a reporter gene and further processed (zebrafish, chick, mouse). Spatial organisation is lost during the process.

2- Barcoding

Each cell's complimentary DNA (cDNA) is identified by a unique barcode for later demultiplexing.

3- Sequencing and associated quality controls

Pooled cDNAs are sequenced using next-generation massively parallel sequencing. Sequences are filtered by several quality control steps, aligned on the reference genome for each cell, and a count matrix is generated. Cells with sufficient count numbers are selected for further analysis.

4- Clustering

The depth of sequencing using one cell is very limited. Most expressed genes are not captured. Compared to bulk sequencing (reading over 90% of the transcriptome), single cell transcriptomes barely cover 10% of the expressed transcripts; for this reason, similar cells are clustered back and *collectively* cover the transcriptome of the population. Each cluster is identified using known markers.

5- Differential gene expression analysis and trajectory analysis are conducted at the level of clusters using a variety of computational methods. For example, check https://hbctraining.github.io/In-depth-NGS-Data-Analysis-Course/sessionIV/lessons/SC_pre- QC.html

**Box 2. Sequencing and genetic approaches**

**Table:** 

Pseudotime	Quantitative method to show the relationship of cells to each other using their transcriptome similarities, thereby showing progression of differentiation in the case of a steady-state system such as differentiation of haematopoietic stem cells used to define this term. In embryos taken at different developmental time points, pseudotime largely matches with developmental time. In neural crest cell populations taken from embryos at one given time point, pseudotime may reflect the wave of anterior–posterior neural crest formation.
Unsupervised clustering methods	The computerized learning process of grouping objects based on similarity without any prior knowledge taken into account
Bar coding	Labelling of a cell’s cDNA that is unique to that cell; cDNA can then be mixed together for sequencing
Genetic labeling	Method to fuse a fluorescent protein/virus/other label to proteins of interest used for cell fate mapping
Gene editing strategies	TALEN (transcription activator like effector nucleases) and CRISPR (clustered regulatory interspaced short palindromic repeats) restriction enzymes for cutting specific DNA sequences, used in genome editing to create mutations
Epistasis	Gene expression of one gene regulated by another

Until recently, most developing systems including the neural crest have been studied at the level of selected cell populations by various well-established "classical" approaches, including fate mapping and gene expression analyses. A cell population is identified *in vivo* by its anatomical position, by the selective expression of marker genes/proteins, or after labelling with a lineage tracer. Alternatively, a cell population can be isolated by microdissection or fluorescence-activated cell sorting (FACS) to be studied *in vitro* or *in vivo*. In contrast, previous transcriptomic and epigenomic analyses have mostly been conducted on whole embryos or large cell populations by "bulk" sequencing of the RNA expression profile of a population: examples include developmental series of whole embryos in *Xenopus*, zebrafish, and mouse^2–4^. More recently, transcriptomes and epigenomes of selected embryonic tissues were isolated by either manual or laser-mediated dissection or using FACS to separate labelled cell groups. These datasets were analysed with or without subsequent spatial reconstruction. Examples include the early mouse embryo^5^, developing blastula-stage half embryos^6^, the gastrula-stage mesoderm^7^, the gastrula and neurula-stage dorsal ectoderm in *Xenopus laevis* embryos^8^, the dorsal neural tube/neural crest in lamprey embryos^9^, or *foxd3-*positive neural crest cells in chick and zebrafish embryos^10,11^. Also using zebrafish, additional bar-coding and temporally controlled gene editing strategies (see Box 2) have opened up the possibility of labelling and subsequently tracing progenitors from early developmental stages to later time points. The results of these experiments have revealed complex cell lineage trees^12,13^.

In this review, we present and discuss the advances of single cell sequencing analyses conducted on neural crest cells in the last 3 years (2017–early 2021). We particularly focus on the two key questions addressed in these studies: the rules of neural crest lineage diversification and the mechanisms driving its multipotency.

## Background

### An overview of neural crest development 

The neural crest is an early multipotent cell population that emigrates from the edge of the closing neural tube during neurulation and organogenesis stages and populates each germ layer of the vertebrate embryo body either directly during early embryogenesis or by the dissemination of derivatives later on (Box 3). Discovered in 1868 by the anatomist Wilhem His as a cell population located between the neural tube and the surface ectoderm and later forming the sensory ganglia, neural crest cells have triggered scientists’ interest ever since.

**Box 3. Embryologic definitions**

**Table:** 

Induction	Ability of a cell to change the fate of an adjacent cell
Classical definition of specification	Capability of a cell to differentiate autonomously when placed in a "neutral" environment
Molecular specification	Expression of genes defined by gene regulatory network for a specific cell type
Determination	Cell capable of differentiating autonomously when challenged by the environment of another region of the embryo
Differentiation	Development of specialized cell types
Derivatives	Cells that arise from a multipotent stem cell, mostly cells that are differentiated
Potency	Potential of cells to differentiate into certain cell types, often tested by removing cell population from the embryo and testing what derivatives are able to form
Canalization	Cell lineage differentiation along defined trajectory that is shaped by the environment and lineage distance
Cell states	Snapshot of time of gene expression in a cell differentiation trajectory
Multilineage priming	The simultaneous activation of several competing developmental programs in multipotent progenitors followed by pruning of competing programs to the benefit of a given fate during commitment

**Figure 1.**
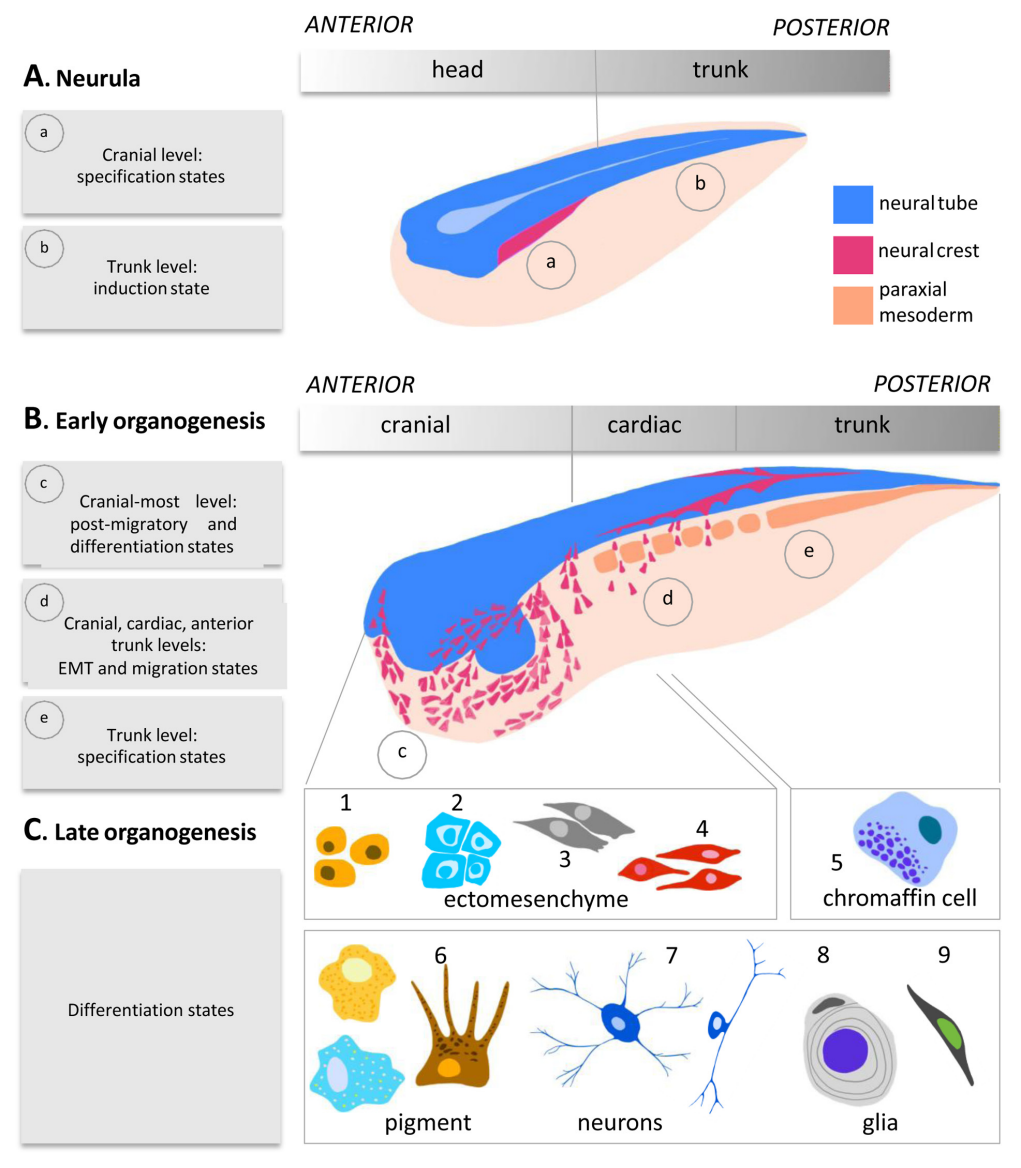
Neural crest states over developmental time. The steps of neural crest development during neurulation and organogenesis are schematised. **A**. During late gastrulation stage (not shown) and neurulation stage, neural crest cells are specified in an anterior-to-posterior wave, with cranial neural crest cells being more mature, e.g. being at specification state (a), while in the trunk, neural crest early induction steps are ongoing (b). **B**. From the end of neurulation to late organogenesis stages, neural crest cells undergo epithelial-to-mesenchyme transition (EMT), migrate, and differentiate. As earlier on, in a given embryo, many neural crest states co-exist. For example, in an early tadpole, anteriorly, cranial neural crest cells finish migration and initiate differentiation (c) while vagal neural crest cells (which include cardiac neural crest cells, at the levels of somite 1 to somite 3–4 in the chick embryo and prospective enteric neural crest cells at the levels of somite 3–8 in chick embryos) undergo EMT and early migration steps (d). In the same embryo, posteriorly, trunk neural crest is being specified dorsal to the neural tube (e). **C**. Last, cranial, cardiac, posterior vagal, and trunk neural crest cells differentiate into specific and common derivatives. Cranial and cardiac neural crest form ectomesenchyme: cranial cells form bone (1), cartilage (2), smooth muscle, and mesenchyme (3), and cardiac neural crest cells contribute to the outflow tract of the heart (4). Trunk-specific fates consist mainly of chromaffin cells in the adrenal medulla (5). At all levels of the anterior–posterior axis, neural crest cells generate pigment cells including melanocytes, xantophores, and iridophores (6), various types of neurons of the sensory, autonomous, and enteric nervous systems (7), Schwann cells and enteric glia (8), and Schwann cell progenitors (9). Neural tissue is depicted in blue, neural crest cells in magenta, and paraxial mesoderm in orange.

Lineage tracing is particularly difficult to complete for the neural crest because of the extensive dispersion of its derivatives across all tissues and organs: rare neural crest-derived cells continue being discovered after 150 years of fate mapping (for a detailed technical and historical review of neural crest fate mapping, please see 14). Collectively, studies conducted in many vertebrate models have unveiled the evolutionarily conserved contribution of the neural crest cells to the peripheral nervous system, including the sensory, sympathetic, autonomous, and enteric neurons and glia, to the various pigment cell lineages with the exception of the pigmented retina, to endocrine cells of the adrenal medulla, to heart outflow tract mesenchyme, and to the mesenchyme and skeleton of the head and neck (Figure 1).

Importantly, neural crest cells contribute to more than 30 different cell types, either directly during early stages of development or by a later contribution of neural crest-derived multipotent progenitors located along the nerves (Schwann cell-associated progenitors^15^). A remaining yet essential gap in our knowledge is the exact pattern of fate decisions *in vivo* resulting in the stereotyped formation of each lineage. Correlated to this essential question, the molecular mechanisms endowing the neural crest with its extraordinary multipotency remain poorly understood, and the few models proposed so far remain open to debate. In this context, a detailed series of single cell transcriptomic and genomic data could inform the existence of neural crest progenitors with progressively more restricted fates.

Traditional fate mapping approaches have described the destiny of spatially defined groups of neural crest progenitors, yielding very precise developmental maps (e.g. 16). Genetic labelling uses selected enhancer or promoter sequences driving reporter expression in subsets of neural crest progenitors (Box 2). Provided that the reporter expression selectively labels the select neural crest cell population throughout development, such fate maps are reliable and have overall confirmed earlier maps proposed from experimental embryology manipulations^17^. In both cases, and in contrast to the data obtained after dissociation into single cells, information on the spatial origin of the lineage is retained.

### The neural crest gene regulatory network

In the last 15 years, a cohort of gain- and loss-of-function studies has set up the scaffold of a complex yet still incomplete network of epistasis regulations between key transcription factors controlling neural crest specification in the gastrula neural/non-neural border ectoderm (induction and specification involve the following genes: *pax3/7, zic1, gbx2, hes4, prdm1a, tfap2a, b, and c, and sox8/9/10*), during neural crest epithelium-to-mesenchymal transition (EMT) at the end of neurulation (specification and EMT involve the following genes: *snail1/2, foxd3, and twist1*), during neural crest migration, and finally during its differentiation during organogenesis (Box 4)^18^. For a more complete review of the neural crest gene regulatory network (NC-GRN) regulators, please refer to 19 for the early GRN at gastrula and neurula stages and to 20 for later stages. More recently, either using candidate gene approaches or by "bulk" sequencing (epigenome and transcriptome), researchers have incorporated numerous novel partners of the network together with their putative transcriptional regulators into the NC-GRN. Examples include a variety of effector molecules such as epigenetic regulators (JmjD2A^21^, DNMT3a/b^22^, and PRDM proteins^23,24^), cell–cell or cell–matrix adhesion molecules and migration regulators (SDF-1 ligand and its receptor Cxcr4^25,26^ and N-cadherin^27^), and novel regulators of signalling pathways (e.g. Axud and Wnt signalling^28^, PFKFB4 and AKT signalling^29^, and Cdon and Shh signalling^30^). Importantly, the NC-GRN is largely conserved throughout vertebrate evolution. As the palette of animal models used in neural crest studies grows, including earlier- and later-derived vertebrates, the logic of evolutionary modulations becomes available on top of the conserved backbone of the NC-GRN^31–34^.

**Box 4. Tentative comparison of neural crest developmental timing between model systems**

*This table uses the earliest time of expression and cranial neural crest area for comparison.

#The timing of melanoblasts (expression of dct) and melanocytes (pigmented cells) differentiation is also compared.

@Human differentiation markers used are neuronal.

**Table:** 

	Neural plate border induction (early gastrulatio n stage)	Neural crest induction (late gastrulatio n stage)	Neural crest specificati on (early–mid neurula stage)	Neural crest epithelium-to-mesenchyme transition (late neurula stage)	Neural crest migration (*early organogenesis stage)	Melanoblast/other differentiation (#)
*Xenopus laevis* (Niewkoop and Faber staging^35^; hours indicated for culture at 23°C)	Stage 10.25 (11 hours); *pax3, msx1, zic1*	Stage 12 (13 hours); *snail2*	Stage 13–16 (15–18 hours); *sox10*, *twist1*	Stage 18–19 (19–20 hours); *sox10*, *twist1*	Stages 19–24* (20–24hours); *sox10*, *twist1*	Stages 26–33 (29–44 hours); *dct*, pigment
Zebrafish (hours post fertilization, [hpf] at 28oC)	70–90% epiboly; 5– 10 hpf; *dlx3b/4b*	11–12 hpf; *tfap2a*, *prdm1a*	12 hpf; *foxd3, snail1b*	13–20 hpf; *sox10, crestin*	20–24 hpf; *sox10, crestin*	36–48 hpf; *mitf, dct*
Chick (Hamburge r and Hamilton [HH] staging^36^; hours at 37°C)	HH stage 3–5 (12–22 hours); *pax7*	HH stage 6 (23–26 hours); *pax7, msx1*	HH stage 6–10*; (23–30 hours); *foxd3, snail2*	HH stage 9–10* (33–38 hours); *snail2, twist1*	HH stage 10–onwards (33–38 hours); *snail2, twist1*	HH stage 13–(48–52 hours); *mitf, dct*
Mouse (Embryonic days post coitum [E]; Theiler Stage [TS]^37^)	E7.75* TS11 *foxc1*	E7.75–8.0* TS12 *tfap2a*	E8.0–8.5* TS12-13 *foxd3,* *sox10*	E8.5–9.25* TS13 *sox10*	E8.5–9.5* TS13 *sox10*	E9.5* TS13–14 *mitf, dct*
Human^38^ (Carnegie stage [CS]^39^)	CS7	CS7–8	CS8	CS11–12 (*Sox9*, *Sox10*, *Pax7*)	CS12 (*Sox9*, *Sox10*, *Pax7*, *AP2a*)	CS12–15 (Sox10, p75^NTR @^)

### What can single cell genomics bring to our understanding of neural crest development?

In the context of the well-advanced NC-GRN, scRNA-seq is expected to confirm the identity of existing neural crest subpopulations, to inform previously unidentified novel groups of neural crest cells, and to provide extended gene signatures for each group of cells. Moreover, single cell genomics will provide evidence for neural crest successive states of development (Box 3) using transcriptome homogeneities among cells of the same cluster (grouped by transcriptome similarities) to infer lineages ("pseudotime", see Box 2) and to identify the root program of cell fate decisions (timing and modalities of fate diversification). In addition, according to the current observation of multilineage priming in various developmental systems (see Box 3 and 40), single cell transcriptomes should identify multipotent progenitors by their specific signature, perhaps including the simultaneous expression of markers for several neural crest-derived lineages^41^. It should be noted that this strategy of inferring a cell's fate using its gene signature at one time point contrasts with the more classical approaches that relied on the actual differentiation of several distinct cell types from a living single cell and thus did not "infer" cell identity but rather cell fate was demonstrated from end-point analysis^42–44^. Furthermore, single cell approaches unavoidably lose the spatial/anatomical information and define groups of cells (clusters) by their expression of known gene markers; this strategy heavily relies on the accuracy of the developmental markers used as a diagnostic of each lineage. In sum, expectations from neural crest-related single cell genomics are high but the limitations can readily be anticipated.

Here, we review recent studies of the neural crest lineage in single cell datasets generated in zebrafish, *Xenopus*, chick, and mouse. Although most of these studies were not designed specifically to study neural crest biology, they provide important insights. After describing a few general features taken collectively from the datasets, we discuss three main points using the model organism best suited to address each of them and then compare the results between species (see a tentative staging comparison between animal models in Box 4): the timing of the neural crest lineage segregation from the other germ layers, the characteristics of neural crest multipotency, and the modalities of neural crest sublineage diversification after EMT. We then finish with a cross-species comparison.

### General features of the neural crest lineage tree viewed from a single cell perspective

The first use of scRNA-seq in zebrafish and *Xenopus tropicalis* were in whole embryos over a variety of stages of development^3,4,45^. As in every single cell study and because of the sparsity of single cell transcriptomes, the first step in single cell data analysis is to cluster cells from the whole embryo into different subpopulations using unsupervised methods (see Box 1 for a simplified standard pipeline of analysis of single cell transcriptome and Box 2 for definitions). Clusters are then characterised by their differential gene expression. Most investigators use a group of known genes that can serve as cell fate anchors for each cell population^18,20^. As discussed above, the accuracy of this information is key to defining cell fates within a lineage. Two separate reports have explored the single cell transcriptomes of whole zebrafish embryos at either 12 time points between high stage and 6 somites (3.33–12 hours post fertilization [hpf]) and 8 stages between sphere and prim5 (4–24 hpf)^4,45 ^respectively. These studies support previous results that suggest that cell fate plasticity and canalisation during differentiation happens in a progressive manner (Box 3). Each population, including neural crest cells, likely uses redundant regulatory signalling and transcriptional programs, and the data from zebrafish suggest that not all cell fates undergo a simple binary decision between two fates but seem to generate more than two lineages with equivalent transcriptome profiles. This is especially evident in Wagner *et al*.’s study (their Figure 2) but also observed in Farrell *et al*., where progenitors can give rise to multiple cell fates of endoderm and non-chordal mesoderm (their Figure 1). In *X. tropicalis*, a large series of 8 successive developmental stages from blastula (stage 8) to early tailbud (stage 22) was investigated by scRNA-seq^3^. Data from this sequencing showed that developmental states do undergo tree-like state transitions, similar to binary decisions but with more possible branches similar to other lineage trees, and that most of the gene expression modules are expressed earlier than previously recognised.

Interestingly, the zebrafish data suggest that the earliest cell fate decisions in the embryo happen within the mesoderm (separating the midline mesoderm notochord and prechordal plate from the rest of the mesoderm lineages), and this is at around the same or at an earlier time point than the separation between different germ layers^45^. In *Xenopus*, however, the first step from the blastula state is the formation of the three embryonic germ layers (ectoderm, mesoderm, and endoderm) along with the separate (pluripotent) germ line at the early gastrula stage^3^ (their Figure 2, stage 10). When single cell transcriptomics is combined with fate mapping of wild-type and mutant embryos, these data suggest that not all zebrafish cell fates have tree-like cell fate trajectories but undergo defined steps of molecular specification^4^ (Box 3). While these appear to be different conclusions than in other models, the differences may be that of timing of the sequencing, the number of time points used in the analysis, and bioinformatics pipelines. The pipelines used in Wagner *et al*. cluster groups mostly based on anterior–posterior information, and these do not always line up with germ layer cell fates and could cause inconsistencies between the datasets. Thus, this pipeline may not pick up previously defined lineage relationships. Furthermore, zebrafish, unlike mice, develop very quickly and as such cell fates may be required to be more dynamic. Interestingly, there is an example of a cell cluster that diverges at 10 hpf, the neural crest cluster from the pharyngeal arches, and then reconverge at 24 hpf, confirming a strong embryonic relationship (their Figure 2). The final overall conclusion from these studies is that mutant cells (*nodal, chordin, tyrosinase*) do not adopt new cell fates on the transcriptional level but express a transcriptional profile similar to that of wild-type cells, suggesting canalisation or a cell fate that is similar to that which wild-type cells would normally differentiate into (Box 3).

## Segregation of the neural crest lineage from the rest of the embryo accompanies its specification

In the zebrafish ectoderm lineage tree, inferred from scRNA-seq trajectories, the neural crest lineage branches close to the spinal cord trajectory, while the neural plate border cells, from which neural crest cells arise *in vivo*, branch away separately^45^ (Wagner *et al*. Figure 1; see discussion on clustering and anterior–posterior differences)^4^. Transcriptionally, tailbud-stage (specified) neural crest cells would be more similar to posterior central nervous system than to neural border cells. In contrast, in *Xenopus*, Briggs and colleagues found that the neural crest lineage branches out early on from the neurectoderm state, itself directly linked to ectoderm^3^. The *Xenopus* data thus detect an earlier transcriptome diversification between neural and neural crest fates than in zebrafish. It would be interesting to directly compare the general neural crest specifier genes and the antero-posterior axis markers that influence data clustering in each study.

Textbook descriptions of neural crest development suggest that specification occurs at the border between neural and non-neural ectoderm at the end of gastrulation or beginning of neurulation. In *X. laevis*, the earliest *snail2* expression is detected at stage 11.5–12 by *in situ* hybridisation and whole embryo bulk RNA sequencing^8,46^, while neural border specification occurs during early gastrulation (stage 10.25)^47–50^. However, recent work has suggested that neural crest specification GRN is set up even earlier at early gastrulation or blastula stages^49–51^. The two latter studies explanted early stage blastula cells and showed that the cells were able to differentiate into neural crest cells autonomously when placed *ex vivo*. Sequencing of FAC-sorted neural crest cells has also been performed in zebrafish and mouse^10,11^. It is now possible to test the idea of the timing of neural crest specification at the level of gene expression (using *bona fide* neural crest specifier expression such as *foxd3* and *snail2*). It seems in *Xenopus* and zebrafish that the specification occurs at mid- to late-gastrulation stages during normal development (stage 12 and 9 hpf, respectively). Moreover, this idea was supported by neural crest cell sorting prior to sequencing using regulatory elements driving expression of the neural crest specifier *foxd3* in zebrafish at 4 stages (75%, 1–2 somites, 5–6 somites, and 14–16 hpf). Interestingly, sorting and sequencing in *foxd3* mutants suggests that the GRN is very interactive, with *foxd3* both activating the GRN of neural crest and repressing other genes in a likely mutual regulatory circuit^10,11^. Together, these data suggest that by selected gene expression analysis, the activation of the current NC-GRN occurs at the time of gastrulation rather than at the blastula stage. However, it would be important to further identify the gene regulatory circuits underlying the observations of early determination in the chick embryo blastula to compare to data from other species.

## Are neural crest cells pluripotent blastula cells in disguise or multipotent neurectoderm-derived progenitors?

**Figure 2.**
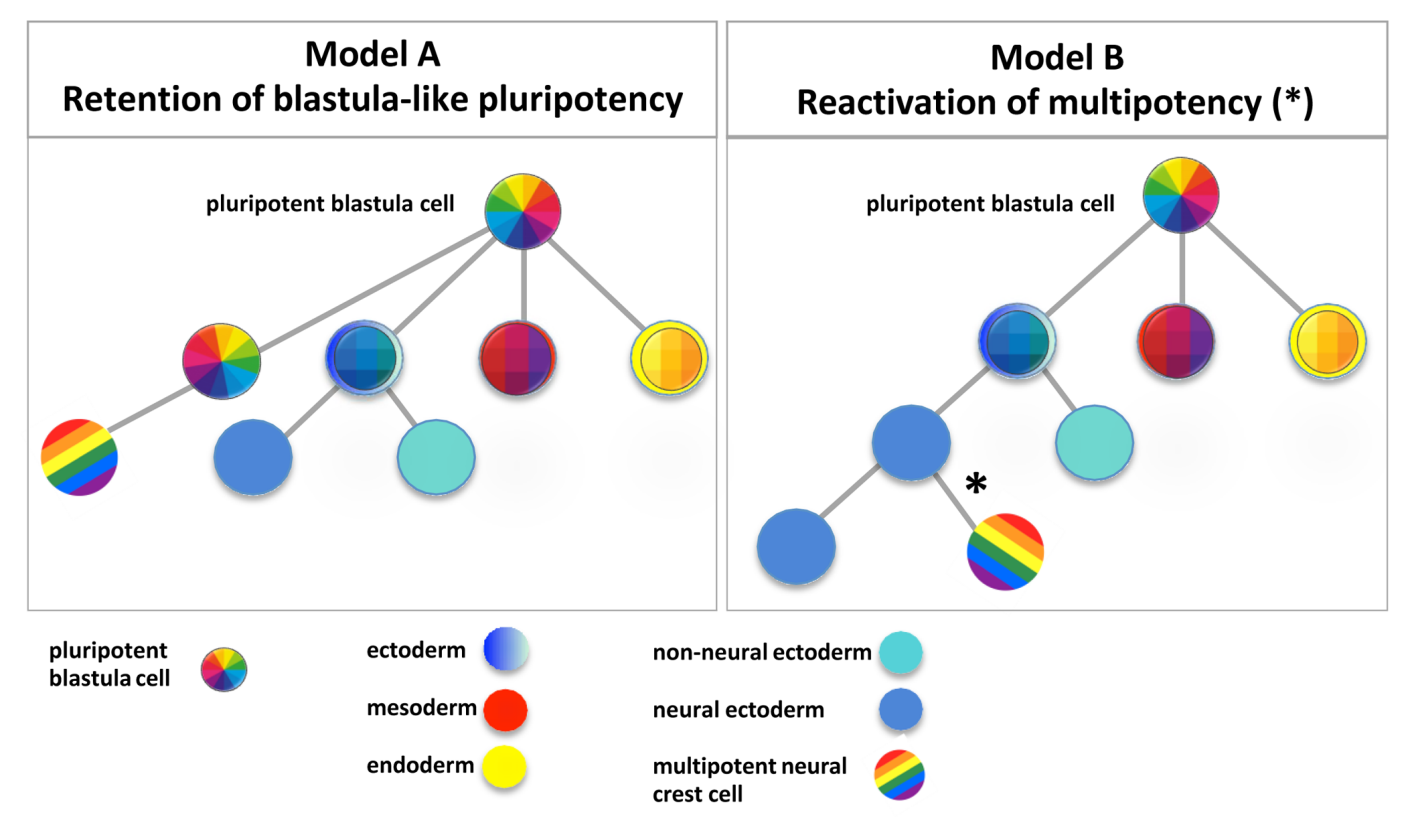
Proposed models of neural crest pluri/multipotency. Two models are proposed to explain neural crest multipotency. **A** In this model, the neural crest lineage shares and retains a specific blastula-type gene signature of pluripotency (rainbow quadrants) while the three other germ layers are segregated into ectoderm (blue), mesoderm (red), or endoderm (yellow). Further subdivision of the ectoderm into neuroectoderm (sky blue) and non-neural ectoderm (turquoise) is indicated. **B**. In this model, the neural crest lineage re-activates (*) pluri/multipotency genes at the neural border (rainbow stripes) after neural lineage segregation (blue, red, and yellow as above).

A major outcome of single cell genomics would be to highlight the molecular basis of neural crest cells’ exceptional multipotency (see 52 for a new term, "pleistopotency", from the Greek "pleistos" ["very large"], proposed to describe the neural crest’s highly diverse potential). Traditional fate mapping and lineage tracing that labels single or groups of cells has established neural crest cells as a multipotent population of cells at the time of specification^53–55^, while other studies have shown that some populations are more restricted^56–59^. *In vitro*, the clonal analysis of single neural crest cells cultured in the presence of various cytokines has provided a fate lineage tree of the chick cranial neural crest, demonstrating multiple degrees of potency, from progenitors restricted to a unique fate to the multipotent cells giving rise to all known neural crest derivatives (42 and references therein). More recently, multipotent neural crest progenitors have been traced individually in the dorsal neural tube *in vivo,* together with some lineage-restricted progenitors in the mouse and in the chick, without any noticeable fate-specific spatial organisation^43,44,60^. Single cell multiplexed *in situ* hybridisation detected the expression of genes related to embryonic pluripotency such as *nanog, klf4,* and *oct4/POUV,* in chick dorsal neural tube progenitors^41^. Interestingly, a detailed examination of the evolutionary history of *oct4/POUV* and *nanog/Ventx* genes showed that those two families arose specifically in vertebrates and may have accompanied the formation of vertebrate-specific cell lineages such as the neural crest^33^. However, the molecular basis of neural crest "pleistopotency" remains a matter of debate. A first model proposed that the neural crest multipotency state retained a gene signature of pluripotency, common between blastula cells and neural crest progenitors at the neural–non-neural border^61^ (Figure 2, Model A). In this study, genes classically associated with neural crest fate in *Xenopus*, such as *snail1* and *sox5,* were found to be expressed in blastula pluripotent ectodermal cells as well. It should be noted here that, as in *Drosophila,* vertebrate *snail1* is also involved in mesoderm development. The participation of several genes involved in neural crest development in other developmental programs is common, such as for *snail2, twist1*, and *foxd3* during mesoderm formation^62–67^. However, this shared expression does not necessarily imply a link with multipotency but rather the multiple functions of a given transcription factor in different developmental contexts. Buitrago-Delgado *et al*. further identified additional genes such as *tfap2a, ets1*,** and *foxd3* shared between neural crest cells and blastula stem cells. Interestingly, they showed that the experimental depletion of *snail1* or *sox5* blocked the expression of pluripotency genes in *Xenopus* blastulas. Lastly*,* neural crest progenitors were challenged to form other germ layer fates by treatment with low or high activin doses *in vitro*. In this case, neural crest progenitors activate markers of mesoderm (*brachyury*, *myod*) and endoderm (*endodermin*), demonstrating that neural crest cells can express markers of the three germ layers and are thus truly *pluripotent*. From these similarities, this study proposed that neural crest progenitors retain a genetic signature controlling pluripotency, suggesting a model in which a subset of cells at the neural border remain similar to blastula cells and pluripotent, while the rest of the germ layers become more restricted and, in the case of dorsal ectoderm, adopt a neuroectodermal fate^61^. This model contrasts with the classic germ layer model (Figure 2, Model B) that proposes that neural crest progenitors arise from neuroectoderm, and thus present with an ectoderm-restricted potential first, and re-activate multipotency features at the neural/non-neural border in a second step. The second model was further supported by the recent functional analysis of the Nanog ortholog Ventx2 in *Xenopus* embryos^33^. Scerbo and Monsoro-Burq showed that if the early expression of Ventx2 is experimentally sustained from late blastula stage until neurula stage, the expression of blastula-specific pluripotency genes is maintained but this *prevents* neural crest formation. In contrast, when activated during gastrulation, i.e. *after* neural border formation has been initiated, *Xenopus* Ventx2 or mouse Nanog are required to elicit multipotent neural crest formation. In order to challenge and explore further these two models, scRNA-seq analyses conducted in different vertebrate and chordate species will allow for a fine-grained analysis of the gene signature(s) of pluripotency/pleistopotency/multipotency and the ability to follow the developmental lineage of the multipotent progenitors and highlighting the evolutionary appearance of multipotent neural crest cells.

Data from scRNA-sequencing in *X. tropicalis* embryos explored the hypothesis that neural crest cells have a shared transcriptional program with blastula pluripotent cells (Figure 2, Model A), an intriguing hypothesis because maintaining the expression of stem cell pluripotent genes is one way to explain why neural crest cells are able to become many different types of cells^61^. This new idea challenged the neuroectoderm hypothesis (Figure 2, Model B), in that neural crest cells arise from the ectoderm lineage and transition through a neural plate border fate to be specified into neural crest cells. Despite a very large number of cells sampled from several closely related gastrula stages (stage 10 whole embryos, dorsal halves of embryos at stage 11, and stage 12 whole embryos), there was no evidence of pluripotency retention during neural crest cell specification, nor did single cell transcriptomes detect a specific relationship between blastula cells and neural crest progenitors. Rather, the gene signature shared between neural crest cells and blastula cells was broadly distributed in many cell types (3 and their Figure 7).

Moreover, recent data from mouse cranial neural crest cells^68^ explored a similar question and strengthened the observation made in *Xenopus* that the neural crest progenitors re-activate the expression of pluripotency genes after neural border specification^33^. Zalc and colleagues sequenced mouse cranial-most neural crest cells during their specification from E7.5 to E8.75. Using single cell transcriptomics, they found that the dorsal neural tube/premigratory neural crest progenitors (Wnt1-positive cells) strongly express *Oct4* and, at lower levels, *Nanog* at the 4-somite stage (E8.0). They showed that *in vivo* the early neural folds do not express *Oct4* (stage E7.75) but that *Oct4* expression is activated in the neural folds at a later stage. By genetic labelling and genetic ablation studies, they further demonstrated that *Oct4-*positive cells generate craniofacial neural crest cells with an ectomesenchyme bias: *Oct4* depletion affects ectomesenchyme derivatives but not neuro-glial fates, similar to mechanisms observed for nanog/ventx2 activity in other species^33,68^.

The single cell data reported from *Xenopus* and mouse is strong evidence that neural crest cells transition through neuroectoderm fate at the time of specification and re-activate a pluripotency circuit involving factors such as Oct4/PouV and Nanog/Ventx. Additional supporting preliminary data from mouse suggest that some genes, but not all of the pluripotent network, function in neural crest cells (Keuls and Parchem, personal communication). Altogether, data from the new single cell sequencing approach more likely support the classical neurectoderm hypothesis (Figure 2, Model B).

## Neural crest cell fate decisions and diversification

How neural crest progenitors diversify into the multiple different cell types is still an open question in neural crest biology. Cell autonomous information enables the cells to have a certain developmental potential, but environmental factors during migration and at the termination of migration are also important for differentiation. Moreover, multiplexed immunostaining or *in situ* hybridisation have detected co-expression of various markers indicative of later fates, suggesting multilineage priming in those cells^53^. Hints of this were observed by sequencing the neural crest cell population by bulk RNA-seq^8,18,69–72^. scRNA- seq holds promise for identifying the gene expression profiles as the cells diversify and indicate if and when they remain a homogeneous population or a mix of heterogeneous cells. However, there are challenges owing to the loss of spatial and positional information along the differentiation trajectory. In addition, increasing the number of time points separated by short time frames will be important, depending on the species, for a better definition of the dynamics of neural crest diversification.

### Diversification of the neural crest in non-amniote aquatic species

In both *Xenopus* and zebrafish datasets^3,4^, the neural crest clusters are very homogeneous during neurulation and separate into several branches only after EMT, during late neurula and tailbud stages (18–24 hpf in zebrafish, stages 18–20–22 in *Xenopus;* Box 4). These sublineages correspond to the three main streams of cranial, cardiac, and trunk neural crest cells. This contrasts with other cell lineages, such as placodes, non-neural ectoderm, or mesoderm derivatives, that exhibit multiple subdivisions corresponding to their early differentiation into different structures. Thus, the single cell transcriptomes detect the coarse- grained neural crest subdivisions at tailbud stage but no finer subdivisions. These data suggest that at transcriptome scale, the neural crest cell population remains highly homogeneous until migration stages (Figure 1B).

### Diversification of the neural crest in amniotes

Less is known about neural crest specification in mammals, partially because of the *in utero* developing environment but also because tools to label neural crest with Cre recombination often are not expressed sufficiently at early enough time points. Recent work from the Adameyko and Wysocka groups use Sox10-Cre and Wnt1-Cre to examine the transcriptome of mouse single neural crest at E7.75, E8, E8.5, and E9.5^68,73^. These studies use a similar sequencing technology and strategy and thus are interesting to compare. At early stages, they capture pre-EMT neural crest precursors in the neural tube. Interestingly, in mouse, these cells express both markers of neural plate border specifiers and neural tube. This continued expression of some neural plate border and neural tube markers suggests that the activation of the neural crest GRN co-exists with dorsal neural tube programs until E9.5 and continues to be expressed after they leave the neural tube. This is in contrast to what is thought in the other models, where the expression of neural crest specifiers is observed before or concurrent with emigration, together with the extinction of neural markers such as *sox2*. The larger dataset from Zalc and colleagues identified four main Wnt1-positive premigratory populations according to their pluripotency status and their anterior–posterior positional signature. They also found that upon EMT, all cranial neural crest cells share a similar transcriptomic signature and diversify after emigration^68^.

In both studies, migrating precursors are the second main neural crest population identified, but the lineage trees inferred from these two closely related studies are not identical. In both, migratory neural crest cells begin to express cell fate-specific factors. In the Zalc study, neural crest cell transcriptomes formed four main clusters: ectomesenchyme, neuroglial, endothelial, and cardiac. In contrast, in the Soldatov study, as they differentiated, the neural crest cells expressed gene networks of specific neural crest derivatives with bifurcations as binary fate restrictions. The first large bifurcation separated sensory from autonomic/mesenchymal branch and then autonomic nervous system from mesenchymal and glia populations. These data further suggest that all neural crest cells express Neurog2 that actively represses melanocyte fate during migration, and cells express two modules simultaneously before solidifying one fate, thereby maintaining a coactivation of alternative fates. While this is consistent with how other cell types differentiate, this is a novel idea for neural crest biology. There is then a cell fate bias in migratory progenitors pushing one fate verses the other. Whether this bias is fully cell autonomous or not is still up for debate, as environmental and/or mechanical cues are also important for neural crest cell fate diversification. In addition to neural crest cells beginning to express several transcriptional programs at the time of delamination, cranial and trunk neural crest share common modules and both begin to activate specific cell fates at the time of delamination. At that time point, the activation of different transcription factors including Twist1 will dictate a specific fate, in this case to ectomesenchymal fates. Overexpression of Twist1 alone is necessary and sufficient to activate a mesenchymal fate transcriptional program in trunk neural crest cells that will never form these derivatives in *vivo*. Interestingly, the reactivation of pluripotency genes in early *Xenopus* neural border progenitors specifically controls *twist1* expression and ectomesenchyme formation *in vivo*^33^. In chick embryos, similar fate specification modules are present. Additionally, many new players can now be inserted into the NC-GRN. For example, hindbrain neural crest cells express neural crest specifiers, as well as chromatin modifiers such as Hmga1, missed in previous bulk RNA-seq analysis^74^ but known to be important for neural crest specification in *Xenopus*^75^. However, there is still a gap in our knowledge regarding how a specific cell fate is activated at the right place and time for the formation of proper derivatives.

## A general cross-species comparison

Interestingly, when the whole embryo *Xenopus* data are compared to zebrafish, there is a high percentage of shared cell fates (79–83%), i.e. hatching gland cells that are unique to fishes and frogs. In addition, the specific lineages identified have multiple shared intermediate states. For example, both species have a population of progenitors that give rise to neural crest-derived xantophores, but they differentiate at different time points in development. Elegant single cell sequencing analysis of post embryonic and adult zebrafish pigment cells suggests that progenitors remain in adults and that thyroid hormone promotes xantophore and melanocyte maturation but in different ways^76^. Moreover, unique cell populations were also identified for each species: for example, the specialized epidermal cells arising in *Xenopus* embryos. Perhaps not surprisingly, there is a conserved reuse of transcription factor modules during development. Some of these factors, including *foxd3* in the neural crest, are expressed within a lineage to define cell fate as a master regulator. Foxd3 both activates and represses gene expression within this lineage and thus has “bimodal” activity in the regulation of neural crest specification and later differentiation^10^. Similarly, in chick and mouse, Foxd3 regulates multipotency during specification and later promotes specific fates (neurons, glia) and inhibits others (pigment)^77–81^. However, Foxd3 and other regulators are often expressed in more than one cell lineage, suggesting the importance of combinatorial reuse of these factors. In terms of evolution, scRNA-seq may also be able to give us clues about the relationships between cells in different organisms. Recent work performed in the ascidian *Ciona intestinalis* examined the expression of genes including *Galanin* in bipolar neurons that is also expressed in a neural crest derivative, dorsal root ganglion neurons, supporting previous work showing that these urochordate neurons arise from the neural plate border and migrate in a similar manner to neural crest cells^82,83^. Together these studies of whole embryo and neural crest-selected scRNA-seq give us clues to how overall cell fate is specified informing neural crest cell specification in general and how they arose during evolution.

## Summary and perspectives

In this review, we have addressed how the innovative single cell approaches available so far inform and add to the knowledge base of neural crest cell specification, multipotency, and diversification. Reassuringly, the single cell data confirm the current understanding of the NC-GRN. However, the thorough analysis of migration and differentiation steps remains to be explored, as well as the analysis of the earliest timepoints of neural crest specification, despite a few important studies on specific neural crest subpopulations. Importantly, these new approaches allow for a comparison of transcriptomes across species and hopefully will resolve long-standing questions regarding neural crest cell specification, multipotency vs. pluripotency, and diversification. Current scRNA-seq along with other experimental studies support the classic hypothesis that neural crest cells arise from neuroectoderm and regain multipotency rather than maintaining a blastula-type pluripotency. However, there are still outstanding questions and technical limitations linked to the completed analyses. For one, scRNA-seq cannot resolve spatial relationships between cell populations. These data would have to be combined with more classical lineage tracing and fate mapping or by new exciting single cell approaches that map sequencing back to spatial expression^84^. Second, for the most part, the sequencing by this method is very shallow, meaning that this approach resolves only the most highly expressed transcripts within neural crest cells from which we are proposing broad and general conclusions. This limited sequencing depth prevents evaluation of the low-level activation of gene expression, which is a critical factor for the understanding of the branch points of the NC-GRN. Additional approaches using nuclear nascent RNA sequencing or transcription at the enhancer sites may nicely complement scRNA-seq and better highlight the subtle dynamics of gene activation^72^. And, third, scRNA-seq has not yet been used to inform how the signalling response is integrated with targeted gene expression in individual cells or by nature and does not resolve important environmental cues that are required for neural crest specification. Specific to neural crest development, future work should 1) increase sampling time points for scRNA-seq to closely capture the intermediate states of the highly dynamic NC-GRN, 2) explore if cell death and elimination between two states influences the neural crest lineage tree by analysis of the apoptotic cascade, and 3) expand the scRNA-seq analysis from neurulation to differentiation time points and include more derivatives during neural crest development. Along these lines, the recent exploration of mouse cranial-most neural crest, using 4 successive stages between 4- and 10-somite stage, has provided additional insights into premigratory and migratory neural crest patterning and pluripotency^68^. All together, we believe that the future is bright. With improvement of the depth of sequencing, integration with spatial expression, and novel bioinformatic approaches, scRNAseq will continue to greatly enhance our understanding of neural crest biology.
